# Osteopathic Manipulative Treatment as a Therapeutic Approach for Fibromyalgia: A Systematic Review

**DOI:** 10.7759/cureus.117441

**Published:** 2026-10-04

**Authors:** Malik El-husari, Ledio Gjunkshi, Abdur-Rehman Hussain, Muhammad Yamaan Khan, Saaketh Kyathari, Julio Mendez, Eden Mohabber, Nadiya A Persaud, Linda Brecher

**Affiliations:** 1 Medicine, Orlando College of Osteopathic Medicine, Winter Garden, USA; 2 Public Health, University of South Florida, Tampa, USA

**Keywords:** chronic pain, fibromyalgia, muscle energy, myofascial release, osteopathic manipulative treatment

## Abstract

Fibromyalgia is a chronic pain disorder characterized by chronic widespread musculoskeletal pain, fatigue, and functional impairment that often remains difficult to manage with conventional therapies alone. Osteopathic manipulative treatment (OMT) is a hands-on, non-pharmacologic approach designed to address somatic dysfunction, but its clinical value in fibromyalgia remains incompletely established. This systematic review examined the association between OMT and pain and functional outcomes in adults with fibromyalgia. A literature search of a single database (PubMed) was conducted using predefined eligibility criteria informed by Preferred Reporting Items for Systematic Reviews and Meta-Analyses (PRISMA) guidance, although the protocol was not prospectively registered and no formal risk of bias assessment was performed. Six studies involving a total of 258 patients were included. The most frequently studied techniques were myofascial release and muscle energy. Across studies, most reported within-group reductions in pain intensity and improvements in functional status or quality of life, but between-group differences were demonstrated in only two studies; treatment protocols ranged from two sessions to 10 sessions over 12 weeks, the longest assessment point was 180 days, and treatment was delivered by physiotherapists or by non-physician osteopaths in five of six studies. Findings were synthesized narratively, as the heterogeneity of study designs, interventions, practitioners, and outcome reporting precluded meta-analysis. However, the certainty of this evidence is low because of the small number of studies, small sample sizes, heterogeneous designs, and the absence of a formal risk of bias assessment. Because most included studies allowed continued pharmacologic care and only one compared medication with and without OMT, the adjunctive contribution of OMT cannot be separated from that of concurrent drug therapy. These findings should therefore be interpreted as a preliminary, hypothesis-generating signal of potential benefit rather than confirmed evidence of effectiveness. Larger and more rigorous clinical trials with standardized risk of bias assessment are needed to confirm whether OMT is effective for fibromyalgia and to define its role in long-term care.

## Introduction and background

Fibromyalgia is a chronic musculoskeletal disorder characterized by chronic, widespread musculoskeletal pain and central sensitization of unknown origin [1,2]. In the United States, fibromyalgia affects approximately 3% of the population and occurs more frequently in females than males at a ratio of 2:1 [2]. The condition is associated with substantial functional impairment and reduced quality of life. Because the underlying pathophysiology remains incompletely understood, treatment is largely symptom-based and focused primarily on pain management. Current approaches typically involve a combination of pharmacologic and non-pharmacologic therapies, including physical therapy, cognitive behavioral therapy, nutritional management, and sleep hygiene interventions [2]. Sleep hygiene consists of behavioral and environmental practices that support restorative sleep and may help mitigate symptom severity [3]. Pharmacologic management commonly includes tricyclic antidepressants, selective norepinephrine reuptake inhibitors, and, in some cases, opioid medications [2].

Osteopathic medicine, founded in 1874 by Dr. Andrew Taylor Still, emphasizes a holistic approach to health through its four core tenets, which recognize the unity of body, mind, and spirit; the body's capacity for self-regulation and self-healing; the reciprocal relationship between structure and function; and the application of rational treatment based on these principles [4,5]. These tenets form the conceptual framework for osteopathic manipulative treatment (OMT), a hands-on therapeutic modality used to address somatic dysfunction. Somatic dysfunction is defined as impaired or altered function of components of the somatic system, including skeletal, arthrodial, and myofascial structures and their related vascular, lymphatic, and neural elements. OMT aims to support the body's inherent capacity for self-regulation [6].

Terminology used in this review

Several terms used throughout this review are specific to osteopathic practice or are used differently across professions, and are defined here for readers outside the osteopathic profession. Osteopathic manipulative treatment (OMT) and osteopathic manipulative medicine (OMM) are used interchangeably in the literature; OMM more broadly denotes the clinical discipline, while OMT denotes the manual treatment itself [6,7]. In the United States, OMT is performed by osteopathic physicians (DOs) and by osteopathic medical students under supervision. Outside the United States, practitioners described as osteopaths are generally non-physician manual therapists whose training and scope differ substantially from those of US DOs [7]. This distinction matters for the present review because most included studies were conducted outside the US osteopathic physician model [8-12].

Muscle energy is a direct, active osteopathic technique in which the patient voluntarily contracts a specific muscle in a precisely controlled direction and intensity against a counterforce applied by the clinician, typically from a position of restrictive barrier engagement, to improve joint motion and reduce muscular hypertonicity [13]. Although the technique originated within osteopathic medicine and is a required component of osteopathic training, essentially the same maneuver is taught and practiced by physical therapists and other manual therapists under the term muscle energy technique (MET); one of the studies included here applied muscle energy in a physiotherapy and rehabilitation setting [12]. It is therefore not exclusive to osteopathic practice, and this review considered studies of MET performed by physiotherapists, provided the technique matched the osteopathic description.

Myofascial release applies sustained pressure and directional loading to restricted soft tissue and fascia with the intent of improving fascial mobility and decreasing pain [1,7]. Neither osteopathic medicine nor physical therapy owns this technique. Myofascial release is used across manual therapy professions, including physical therapy, chiropractic, and massage therapy, and the myofascial release applied within OMT is best understood as a subset of myofascial release as a whole; the myofascial release arm of Sánchez et al., for example, was delivered by physiotherapists [7,9,11]. What distinguishes the osteopathic application is not the maneuver but the framework around it; technique selection follows an osteopathic structural examination that identifies specific somatic dysfunction, the treatment is individualized to those findings rather than applied as a fixed protocol, and it is integrated with the broader medical management of the patient. Because the hands-on procedure is largely shared, findings from myofascial release trials conducted by non-osteopathic practitioners are informative for OMT but are not directly interchangeable with it.

Two interventions appearing in the included studies are not osteopathic techniques and are described here to avoid confusion. Gentle touch therapy, as studied by Salgado et al., refers to micro-physiotherapy as follows: a very low-force manual technique in which the practitioner palpates superficial and deep tissues, identifies restrictions in tissue mobility, and applies small approximation and separation movements, largely at the level of the skin, following a dermal map of embryologic muscle correspondence [11]. It is not massage and is not part of the osteopathic technique set. Dry needling, the active comparator in Sánchez et al., is an invasive procedure in which a solid filiform needle is inserted directly into a myofascial trigger point to reduce local and referred pain; it is outside the scope of OMT and appears in this review only as a comparator against myofascial release [9].

While Dr. Still developed the foundational basis and principles of osteopathic medicine and treatment modalities, subsequent advancements in treatment technique have expanded the tools available to clinicians managing musculoskeletal dysfunction [4,5]. These modalities have been proposed as adjuncts for musculoskeletal dysfunction and functional impairment in patients with chronic pain conditions such as fibromyalgia.

Evidence suggests that multimodal treatment strategies may provide superior long-term outcomes for individuals with fibromyalgia [2,7-12,14,15]. Osteopathic manipulative medicine (OMM) has been proposed as a non-pharmacologic or adjunctive therapeutic option consistent with this multimodal approach, and may be of particular interest to patients seeking alternatives to medication-based management. However, prior literature addressing manual and complementary therapies in fibromyalgia has largely evaluated heterogeneous mixtures of modalities (e.g., massage, acupuncture, physical therapy) rather than isolating osteopathic technique specifically, and no recent systematic review has consolidated the PubMed-indexed evidence evaluating OMT/OMM alone against standardized pain visual analog scale (VAS) and functional Fibromyalgia Impact Questionnaire (FIQ) outcomes in adults with fibromyalgia. This represents a gap this review sought to address.

This systematic review aimed to evaluate the association between osteopathic manipulative treatment and pain and functional outcomes in adults with fibromyalgia, using a framework structured around Population, Intervention, Comparison, and Outcome (PICO) as follows: adults diagnosed with fibromyalgia (Population); osteopathic manipulative treatment or osteopathic manipulative medicine techniques (Intervention); usual care, pharmacologic therapy, alternative manual therapy, or no intervention, where reported (Comparison); and pain intensity measured by the visual analog scale (VAS) and functional status measured by the Fibromyalgia Impact Questionnaire (FIQ), with quality of life as a secondary outcome (Outcome) [16,17].

## Review

Methods

Research Question

Is osteopathic manipulative treatment (OMT) associated with improved outcomes in the management of fibromyalgia, as measured by Fibromyalgia Impact Questionnaire (FIQ) scores and pain intensity assessed using the visual analog scale (VAS)?

Search Strategy

This systematic review was conducted using a structured literature search approach informed by PRISMA principles, although a formal protocol was not prospectively registered (e.g., with the International Prospective Register of Systematic Reviews {PROSPERO}); this is acknowledged as a limitation below [17]. A literature search was performed in PubMed using predefined eligibility criteria. Boolean operators were applied to refine the search strategy, and the following search strings were used: (“osteopathic manipulation” AND “chronic pain”), (“fibromyalgia” AND “osteopathic manipulative medicine”), and (“fibromyalgia” AND “osteopathic treatment”). Duplicate records and studies unrelated to fibromyalgia or osteopathic manipulative medicine (OMM) were excluded using Covidence software (Melbourne, Australia: Veritas Health Innovation Ltd.). The search was restricted to a single database (PubMed) and to a limited set of search strings, primarily reflecting the resource and time constraints of the review team. This is a substantive limitation as it may not have captured all relevant studies, particularly those indexed only in Embase, Cochrane Library, or other databases, and may have reduced the sensitivity of the search. Because no Medical Subject Headings strategy was used and the search terms were restricted to three Boolean strings, the search should be regarded as a focused sample of the PubMed-indexed literature rather than an exhaustive one, and the review is accordingly presented as an evidence map rather than as a definitive synthesis.

Eligibility Criteria

The study population included adult male and female patients diagnosed with fibromyalgia according to the American College of Rheumatology diagnostic criteria [18]. Eligible study designs included randomized controlled trials, prospective studies, retrospective studies, and other observational or pre-/post-intervention study designs. The primary interventions of interest were OMM techniques, including but not limited to muscle energy and myofascial release. Because muscle energy and myofascial release are practiced across several manual therapy professions, studies were eligible when the technique studied matched the osteopathic description of that technique, regardless of the discipline of the practitioner delivering it; the practitioner type was extracted and reported for every included study so that applicability to osteopathic physician practice can be judged directly. Outcomes were evaluated using FIQ scores and pain severity measured by VAS. Only articles published in English between 2015 and 2025 were considered for inclusion; this restriction primarily reflected translation-resource constraints of the review team rather than an a priori judgment that non-English or older literature was less relevant, and is acknowledged as a source of potential language and time-lag bias. Studies were included if they reported clinically relevant outcomes related to pain, function, or quality of life following osteopathic or manual therapy interventions.

Studies were excluded if they did not include a diagnosis of fibromyalgia based on American College of Rheumatology criteria or did not evaluate OMT as an intervention [18]. Ineligible study types included case reports, other systematic reviews, narrative reviews, scoping reviews, and studies involving animal subjects. Articles not published in English or outside the 2015-2025 timeframe were also excluded, as were studies that did not report measurable clinical outcomes (e.g., pain or functional metrics). Non-randomized and observational designs (e.g., retrospective and pre- and post-intervention studies) were eligible for inclusion provided they met the criteria.

Study Selection and Data Extraction

Covidence software (Melbourne, Australia: Veritas Health Innovation Ltd.) was used to manage study screening and selection. Two independent reviewers conducted title and abstract screening to identify potentially eligible studies. Full-text articles were then reviewed by the same independent reviewers to confirm eligibility. Discrepancies were resolved by consultation with a senior author. From included studies, data were extracted on FIQ scores and pain levels measured by VAS to evaluate the effects of OMT across studies. Additional variables, including study design, sample size, intervention type, participant sex, and duration of follow-up, were also extracted. To address the clinical applicability of the findings, the following variables were also extracted from each full-text article: the specific technique or techniques applied and the anatomic regions treated; number, frequency, and length of treatment sessions and the total treatment period; the professional discipline and training of the practitioner delivering treatment; comparator or control condition; any co-interventions, including concurrent pharmacologic therapy; and the timing of every outcome assessment, including the longest post-treatment assessment point. Where a pain scale other than the VAS was used, the instrument actually employed is reported.

Risk of Bias Assessment

A formal risk of bias assessment (e.g., Cochrane Risk of Bias 2 tool for randomized trials or Risk Of Bias In Non-randomized Studies - of Interventions (ROBINS-I) for non-randomized studies) was not performed. This decision reflected the small number of included studies (n=6) and their mixed methodological designs, spanning randomized controlled trials, prospective and retrospective cohort studies, and pre- and post-intervention studies. It is acknowledged as a substantive methodological limitation rather than as a justified omission, and it is the principal reason the present findings are framed as descriptive rather than confirmatory.

Data Synthesis and Reporting Bias

Findings were synthesized narratively rather than pooled quantitatively. A meta-analysis was not performed because the included studies varied substantially in intervention protocol, comparator, outcome timing, and study design, precluding meaningful statistical pooling; consequently, no summary effect sizes, confidence intervals, or heterogeneity statistics (e.g., I^2^) are reported. To minimize the risk of reporting bias, potential sources including publication bias, selective outcome reporting, language bias resulting from the exclusion of non-English studies, and time-lag bias were considered throughout the review process.

Results

A total of 311 records were identified through the PubMed search. After removal of 40 duplicate records and 90 records removed for other reasons, 181 records were screened. Following title and abstract screening, 169 records were excluded for irrelevance. A total of 12 articles progressed to full-text review; of these, six were excluded, and six met all inclusion criteria and were included in the final analysis (Figure 1).

**Figure 1:**
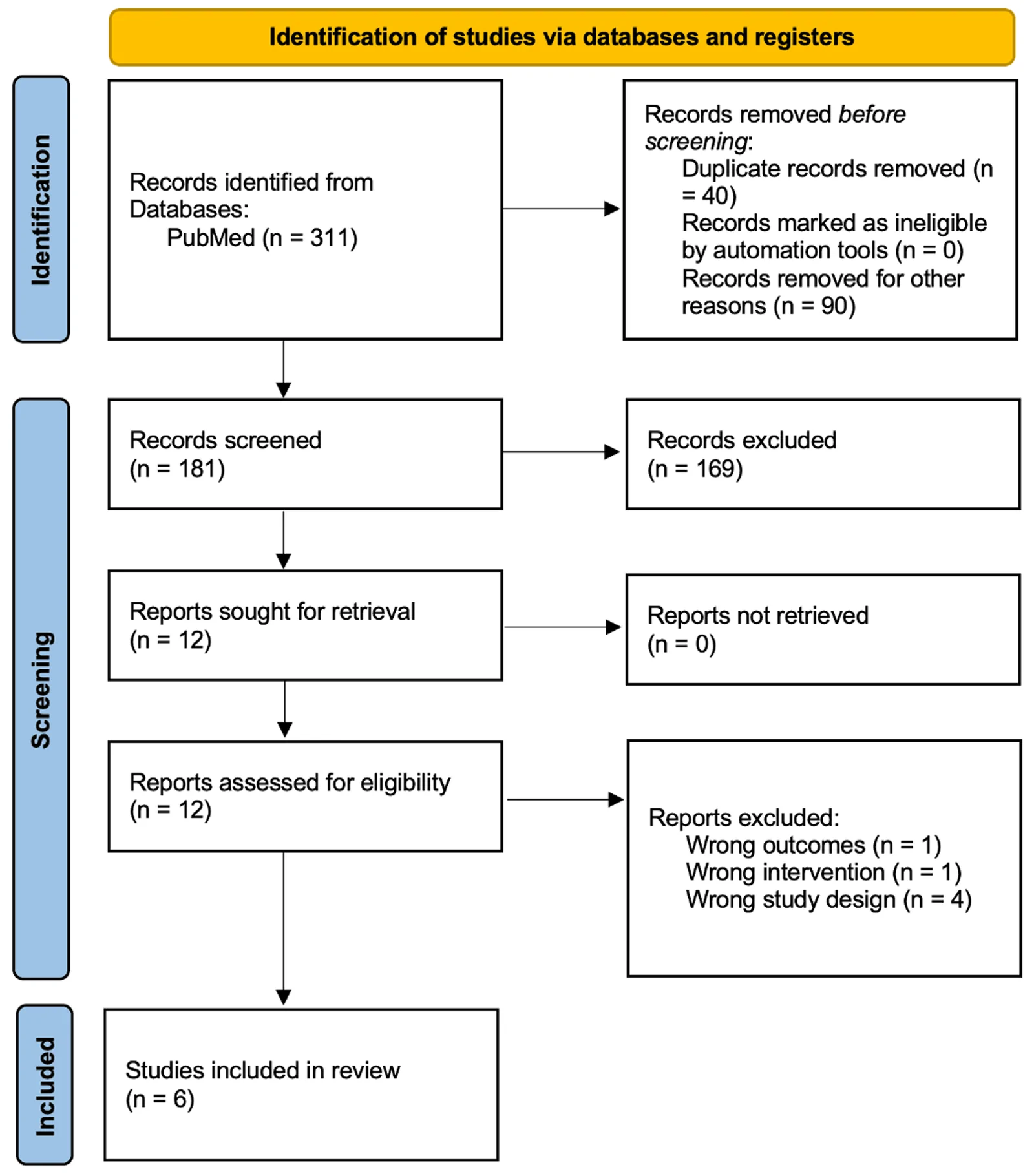
PRISMA flow diagram of study selection.

Across the six included studies, a combined total of 258 patients with fibromyalgia were evaluated (Table 1). Study outcomes were assessed using VAS pain scores and FIQ measures. All included studies reported within-group improvement in pain intensity, fibromyalgia disease impact, or both, but the strength of that signal varied considerably with design [8-12,19]. Only the following two studies demonstrated a statistically significant advantage over a control or sham condition: Albers et al., in which both osteopathic arms improved relative to an untreated control on VAS pain [8], and Salgado et al., in which gentle touch therapy outperformed sham on the area under the pain curve [11]. Two studies had no comparator arm at all; one compared myofascial release against an active invasive comparator without a control arm, and one found no significant between-group differences among its three arms [9,10,12,19]. Notably, no study demonstrated a significant between-group effect on the FIQ, the functional measure specified in this review's research question [8,9,11,19]. The included studies used various methodological designs, including randomized controlled trials, prospective studies, retrospective analyses, and pre- and post-intervention studies. Sample sizes ranged from 14 to 64 participants. The studies are summarized in Table 1, along with the OMT techniques, outcomes, and study-specific limitations. Common methodological limitations across studies included small sample sizes, limited or absent control groups, short follow-up durations, and challenges related to blinding inherent to hands-on therapy trials. Interventions were heterogeneous in both technique and duration, which limits direct comparison across studies and precluded meta-analysis.

**Table 1:** Summary of included studies evaluating the clinical outcomes of osteopathic manipulative treatment for fibromyalgia. OMT techniques assessed across studies included muscle energy and myofascial release. Outcome measures varied by study but commonly included pain intensity, functional status, and disease severity. Sex distribution and, where available, per-arm sample sizes were confirmed against each study's full published article. OMT: osteopathic manipulative treatment; OMM: osteopathic manipulative medicine; FIQ: Fibromyalgia Impact Questionnaire; TP: tender points; OI: overall improvement; GOT: global osteopathic treatment; BDNF: brain-derived neurotrophic factor

Studies	Purpose	Study design	Sample size	Population by sex	OMT technique used	Outcomes	Limitations
Albers et al. [8]	Effect of osteopathic treatment on pain threshold and disease severity in fibromyalgia patients	Randomized controlled trial	50 patients randomized (control n=14; OI n=19; GOT n=17, 1 dropout, 16 analyzed)	2 male, 48 female overall (control: 0 male/14 female; OI: 2 male/17 female; GOT: 0 male/17 female, per published baseline table)	Muscle energy; myofascial release	OI and GOT decreased pain intensity; TP and FIQ not statistically significant	Occurrence of osteopathic dysfunction was not systematically investigated; the planned sample of 60 was not reached due to time and resource constraints, producing group-size imbalances
Farra et al. [10]	Effect of myofascial release on functional status of fibromyalgia patients	Retrospective study	14 patients analyzed (of 21 who originally underwent OMT; 16 met inclusion criteria; 14 had complete outcome data)	All female (n=14), per published article	Myofascial release	Decreased impact of fibromyalgia on functional status; improved quality of life	No control or comparison group; no experimental methodology; small sample size; all-female sample limits generalizability to male patients
Marske et al. [19]	Effect of gabapentin vs. osteopathic treatment in reducing pain in fibromyalgia patients	Prospective study	29 patients analyzed (8 gabapentin only; 11 OMM only; 10 gabapentin+OMM)	2 male, 27 female overall (2 of 11, i.e., 18%, in the OMM-only arm; 0% male in the gabapentin-only and combination arms, per published baseline table)	Muscle energy; myofascial release	Significant pain reduction for OMM only; less significant for OMM+gabapentin; not significant for gabapentin alone	No sample size or power calculation; small treatment groups; authors' own post hoc analysis indicated roughly 21 participants per arm would be needed for adequate power
Salgado et al. [11]	Effect of gentle touch therapy on pain relief and neuroplasticity in fibromyalgia patients	Double-blind randomized controlled trial	64 patients (32 gentle touch therapy; 32 placebo)	All female	Gentle touch therapy	Significant decrease in pain and brain-derived neurotrophic factor	All-female sample limits generalizability to male patients; not possible to be truly double-blinded given hands-on intervention
Sánchez et al. [9]	Effect of dry needling vs. myofascial release on pain, sleep, fatigue, and anxiety in fibromyalgia patients	Single-blind randomized controlled trial	64 patients (32 dry needling; 32 myofascial release)	58 female, 6 male overall (30 female/2 male dry needling; 28 female/4 male myofascial release)	Myofascial release	Dry needling and myofascial release both improved pain	Lack of follow-up assessment; techniques were combined with other modalities
Uysal et al. [12]	Effect of muscle energy on respiratory muscle strength in fibromyalgia patients	Pre- and post-test research study	37 patients	All female	Muscle energy	Increased muscle strength and endurance; decreased pain intensity	Outcomes measured at different times; lack of control group

Treatment Dose, Practitioner, and Duration of Effect

Treatment protocols differed by an order of magnitude across the six studies (Table 2). The most intensive protocol delivered 10 individualized osteopathic sessions over 12 weeks [8]; the least intensive delivered two sessions 45 days apart [11]. Marske et al. provided 30 min of OMM weekly for six weeks; Uysal et al. provided three sessions weekly for three weeks; Sánchez et al. treated over four weeks; and Farra et al. followed patients through a course of OMT delivered over four months [9,10,12,19]. This variation alone would preclude any statement about an effective dose of OMT in fibromyalgia [8-12,19].

**Table 2:** Treatment dose, practitioner, comparator, and longest assessment point in the included studies. OMT: osteopathic manipulative treatment; OMM: osteopathic manipulative medicine

Studies	Who delivered treatment	Treatment dose and duration	Comparator	Longest assessment point
Albers et al. [8]	Osteopaths at a German osteopathy school (non-US-trained osteopathic physicians)	10 treatment sessions over 12 weeks	Untreated control group	End of the 12-week treatment period
Farra et al. [10]	Osteopaths at an Italian osteopathy institute (non-US-trained osteopathic physicians)	Course of OMT sessions over 4 months, in addition to usual care	None (single-arm records review)	One month after the end of treatment (5 months from baseline)
Marske et al. [19]	Advanced medical students at a US college of osteopathic medicine	30 min weekly for 6 weeks	Gabapentin 900 mg/day; gabapentin plus OMM	8 weeks from baseline
Salgado et al. [11]	Physiotherapists trained in micro-physiotherapy	Two sessions of 40-45 min, 45 days apart	Sham (simulated) gentle touch	180 days from baseline
Sánchez et al. [9]	Physiotherapists	Four weeks of intervention	Dry needling (active comparator)	Immediately post-treatment (4 weeks)
Uysal et al. [12]	Clinicians in a physiotherapy and rehabilitation department	3 sessions per week for 3 weeks, after superficial heat	None (single-arm pre- and post-test)	End of the 3-week treatment period

The duration of effect is even less well characterized. Four of six studies assessed outcomes only up to the end of the treatment period, with no post-treatment follow-up, so no statement about persistence of benefit is possible from them [8,9,12,19]. Farra et al. reported that functional gains seen at one month were maintained through a one-month post-treatment follow-up [10]. Salgado et al. provided the only long observation window, following patients for 180 days, and found a between-group difference in cumulative pain scores but no change in FIQ or 36-Item Short Form Health Survey (SF-36) quality of life at any point [11]. The available evidence therefore supports, at most, short-term symptomatic change during or immediately after a treatment course, and does not establish that any benefit persists once treatment stops.

The professional discipline of the treating practitioner also varied and is directly relevant to the applicability of these findings to osteopathic physician practice. Treatment was delivered by physiotherapists in three studies [9,11,12], by osteopaths trained in European non-physician osteopathic programs in two studies [8,10], and by advanced osteopathic medical students under supervision in one [19]. No included study evaluated OMT delivered by practicing osteopathic physicians.

Discussion

Clinical Impact of OMT in Fibromyalgia

Across the six included studies, osteopathic manipulative treatment (OMT) was associated with improvements in pain, functional status, and quality of life among patients with fibromyalgia (Table 1) [8-12,19]. Most studies evaluated musculoskeletal outcomes using validated patient-reported measures, including the visual analog scale (VAS) and Fibromyalgia Impact Questionnaire (FIQ). That association, however, rests largely on within-group change. Controlled comparisons were available in only four studies, and of these only two found a significant advantage over an inactive comparator, neither of which involved OMT delivered by an osteopathic physician [8,9,11,19]. No study found a significant between-group difference in FIQ [8,9,11,19]. Although the overall direction of findings suggests that OMT may provide therapeutic benefit, the available evidence is insufficient to establish effectiveness.

Myofascial release and muscle energy were the most frequently evaluated techniques. Myofascial release is intended to reduce fascial restrictions that may contribute to pain and altered biomechanics, while muscle energy techniques use active patient participation to improve mobility and reduce muscular hypertonicity. The observed improvements in pain, strength, endurance, and functional capacity are consistent with the hypothesis that treatment of somatic dysfunction may influence peripheral nociceptive input and central pain processing. However, the included studies did not directly evaluate neurophysiological or mechanistic outcomes. Therefore, this remains theoretical and warrants further investigation.

Because the manual procedures involved are shared across professions, the studies conducted by physiotherapists should be read as evidence about the techniques rather than about osteopathic care as a whole. Osteopathic practice adds an individualized structural diagnosis and integration with medical management that these trials did not isolate [9,11,12], and none of the included studies compared protocol-driven technique application with osteopathically individualized treatment [8-12,19].

Comparison With Pharmacologic Management

Pharmacologic therapies, including gabapentin and duloxetine, remain common components of fibromyalgia management because they target neuropathic pain pathways and neurotransmitter dysregulation [2]. However, symptom relief may be incomplete, and adverse effects can limit long-term adherence.

Only one included pilot study directly compared medication alone with medication combined with OMT [19]. In that study, both the OMM-only arm (p<0.01) and the combined OMM plus gabapentin arm (p=0.03) improved significantly from baseline on the Wong-Baker FACES pain scale, whereas the gabapentin-only arm did not; the OMM-only arm was also the only group to improve on the Clinical Global Impression of Health scale (p<0.01). Critically, no comparison between the three arms reached statistical significance, and neither FIQ scores nor tender point counts changed significantly in any arm. The study therefore does not show that combined treatment was superior to either component, and it does not establish a synergistic effect. It is best read as a feasibility study demonstrating that both interventions were safe and tolerable at this sample size.

The adjunctive value of OMT relative to pharmacotherapy cannot be isolated from the remaining studies either. Farra et al. delivered OMT in addition to usual care, which included ongoing medication [10]; Salgado et al. enrolled only patients who had received pharmacologic treatment for more than three months without improvement and permitted existing therapies to continue [11]; and the other included studies did not report or control concurrent drug therapy [8,9,12,19]. Consequently, observed changes reflect OMT layered onto an unmeasured background of pharmacologic and non-pharmacologic care rather than the effect of OMT in isolation. Trials that randomize patients to a stable medication regimen with or without OMT, with drug exposure documented throughout, are required before any adjunctive claim can be made.

Fibromyalgia involves multiple interacting mechanisms, including altered pain processing, autonomic dysfunction, and neuroendocrine dysregulation. OMT has been proposed as a multimodal intervention that may address biomechanical, neurologic, and autonomic factors [2,3]. This rationale remains conceptual because these pathways were not directly examined in the included studies.

Osteopathic Principles and Whole-Person Care

Fibromyalgia extends beyond musculoskeletal pain and commonly involves fatigue, sleep disturbance, anxiety, depression, and impaired daily functioning [2,3]. These symptoms contribute substantially to reduced quality of life and support the use of a whole-person approach to management. The osteopathic principle that the body functions as an integrated unit provides a relevant conceptual framework for addressing the multidimensional burden of fibromyalgia.

Several included studies reported improvements in fatigue, anxiety, or functional status in addition to pain [8-12,19]. These findings suggest that the potential effects of OMT may extend beyond pain relief. This signal should be interpreted cautiously, since the two studies with the most rigorous control conditions found no change in validated quality of life instruments as follows: FIQ and SF-36 were unchanged in the study by Salgado et al., and FIQ did not change significantly in the study by Albers et al. or Marske et al. [8,11,19]. However, further research is needed to determine whether OMT directly influences psychological well-being, central pain processing, or other systemic features of fibromyalgia.

Limitations of the Evidence and Certainty of Findings

The available evidence has several important limitations. The six included studies had small and heterogeneous samples, varied methodological designs, and frequently lacked control groups. Differences in OMT techniques, treatment frequency, follow-up duration, and outcome measures further limited comparisons across studies. Three studies included only female participants [10-12], and only 10 of the 258 participants across the included studies were male, which substantially limits generalizability to male patients [8,9,19]. Blinding is also inherently difficult in manual therapy research and may introduce performance and detection bias. A formal risk of bias assessment was not performed, preventing a structured evaluation of internal study validity and reducing confidence in the reported associations. Publication bias was also not formally assessed. The search was limited to PubMed and did not use a comprehensive Medical Subject Headings strategy. The omission of additional databases may have resulted in relevant studies being missed. Eligibility was also restricted to English-language studies published within the previous 10 years, which may have excluded relevant research. Because the findings were synthesized narratively without meta-analysis, effect sizes could not be pooled or compared quantitatively. The review was also not prospectively registered, which limits methodological transparency.

Three further limitations bear directly on how much this review can support. First, the interventions studied were not uniformly osteopathic as follows: one included study evaluated micro-physiotherapy and another used dry needling as its comparator, and no included study evaluated OMT delivered by a practicing osteopathic physician, so applicability to US osteopathic practice is indirect [8,9,11,12,19]. Second, the heterogeneity in design, dose, comparator, practitioner, and outcome timing is severe enough that the included studies cannot be meaningfully combined, and this review should be read as a structured map of a thin and inconsistent evidence base rather than as a source of pooled conclusions (Tables 1, 2). Third, because concurrent pharmacotherapy was permitted and unmeasured in most studies, the independent contribution of OMT cannot be separated from that of drug therapy [8-12,19]. Collectively, these limitations indicate that the evidence is of low certainty and should be interpreted as suggestive of potential benefit rather than confirmation of OMT effectiveness.

Clinical Implications and Future Directions

OMT may represent a promising adjunctive approach for managing fibromyalgia symptoms, particularly among patients who experience inadequate relief or adverse effects from pharmacologic treatment. At present, this is a rationale for further study rather than a finding; the reported improvements in pain, function, and quality of life support continued investigation but do not yet justify definitive clinical recommendations, and clinicians should not infer from this review that OMT has an established effect on fibromyalgia-specific functional outcomes [8-12,19].

Future research should prioritize adequately powered randomized controlled trials with standardized treatment protocols, appropriate comparison groups, longer follow-up periods, and more diverse patient populations. Future systematic reviews should conduct multidatabase searches and apply design-appropriate risk of bias tools. Trials should also report the treatment dose and the discipline and training of the treating practitioner, document concurrent pharmacologic therapy so that adjunctive effects can be isolated, include assessment points well beyond the end of treatment to characterize the duration of any effect, and enroll male participants in meaningful numbers. Mechanistic studies evaluating central sensitization, autonomic function, and neurophysiological responses may clarify how OMT influences fibromyalgia symptoms. Research should also examine cost-effectiveness, accessibility, and the role of OMT within multidisciplinary care.

## Conclusions

This systematic review found that osteopathic manipulative treatment was associated with improvements in pain, functional status, and quality of life across six studies of adults with fibromyalgia, most often following myofascial release and muscle energy technique, which are manual approaches used across multiple disciplines and are not unique to osteopathic practice. These improvements were largely within-group. Only a minority of studies reported controlled between-group differences favoring OMT, and none reached statistical significance on the Fibromyalgia Impact Questionnaire. Reporting of treatment dose, duration of effect, and comparator techniques such as “gentle touch” and “dry needling” was inconsistent across studies. Concurrent pharmacotherapy was generally permitted but not measured, so the specific contribution of OMT cannot be isolated. Combined with the small and heterogeneous evidence base, single-database search, lack of prospective registration, absence of meta-analysis, and absence of a formal risk of bias assessment, these gaps in technique definition and outcome reporting preclude pooling and do not support definitive conclusions about OMT effectiveness for fibromyalgia. The findings should therefore be considered preliminary and hypothesis-generating rather than evidence of established benefit. Future trials should standardize technique definitions and dosing, report practitioner discipline, use controlled comparators, measure concurrent pharmacotherapy, and extend follow-up beyond the treatment period.
